# Immediate versus deferred stenting for patients undergoing primary or emergent percutaneous coronary intervention: Protocol for a systematic review and meta-analysis

**DOI:** 10.1097/MD.0000000000008477

**Published:** 2017-11-27

**Authors:** Yong Liu, Sheikh Mohammed Shariful Islam, Clara K. Chow, Shiqun Chen, Muhammad Umer Siddiqui, Qiang Li, Kai-yang Lin, Kun Wang, Guoli Sun, Ying-ling Zhou, Jiyan Chen, David Brieger

**Affiliations:** a Department of Cardiology, Provincial Key Laboratory of Coronary Heart Disease, Guangdong Cardiovascular Institute, Guangdong General Hospital, Guangdong Academy of Medical Sciences, Guangzhou, China; b The George Institute for Global Health; c University of Sydney, Camperdown, NSW; d Westmead Hospital, Sydney, Australia; e Department of Internal Medicine, Mount Sinai School of Medicine (Englewood) Program, Englewood, NJ; f Concord Repatriation General Hospital, Concord, NSW, Australia.

**Keywords:** myocardial infarction, non-STEMI, percutaneous coronary intervention, STEMI

## Abstract

**Introduction:**

Primary or emergent percutaneous coronary intervention (PCI) with stenting is the standard treatment for patients with ST-segment elevation myocardial infarction (STEMI) or non-STEMI acute coronary syndromes (ACS) at high risk. The value of delayed stenting following balloon-facilitated reperfusion in these patients is largely unknown.

**Methods and analysis:**

This systematic review aims to assess whether delayed stenting (vs immediate stenting) improves angiographic and cardiovascular clinical outcomes for patients with STEMI or non-STEMI ACS undergoing primary or emergent PCI. The primary endpoint is adverse angiographic outcomes (no or slow coronary flow after final PCI), the main secondary endpoint includes a composite of long-term (≥6 months) all-cause mortality, recurrent ACS (recurrent myocardial infarction, unplanned revascularization of the target vessel, etc.), hospital admission for heart failure or any other cardiovascular cause. Relevant studies will be searched in the Cochrane Central Register of Controlled Trials, MEDLINE, EMBASE, and other electronic databases. Two authors will independently screen studies for inclusion, consulting with a third author where necessary to resolve discrepancies. The risk of bias of included studies will be assessed using the Cochrane Collaboration risk of bias tool, and quality of evidence using the Grades of Recommendation, Assessment, Development and Evaluation (GRADE) approach. Results will be presented using risk ratios with 95% confidence interval (CI) for dichotomous outcomes and standardized mean differences with 95% CI for continuous outcomes.

**Ethics and dissemination:**

This systematic review and meta-analysis protocol will not require ethical approval. We will disseminate the findings of this systematic review and meta-analysis via publications in peer-reviewed journals.

## 1 Background

To recover the coronary flow and salvage myocardium, timely primary or emergent percutaneous coronary intervention (PCI) is essential and standard treatment for patients with ST-segment elevation myocardial infarction (STEMI) or non-STEMI acute coronary syndromes (ACS) at high risk.^[1,2]^ However, after wiring and balloon dilatation during primary or emergent PCI, most infarct-related lesions contain residual thrombus which might result in reduced coronary blood flow, distal embolization complications that are associated with impaired prognosis.^[3,4]^ The TOTAL study showed that during primary PCI, manual thrombectomy, as compared to PCI alone, did not reduce the risk of adverse cardiovascular event but was associated with an increased rate of stroke.^[5]^ In addition, immediate stent implantation in the thrombotic environment may provoke immediate microvascular obstruction (MVO) with higher adverse effects despite preventing early postprocedural coronary occlusion and late restenosis.^[6,7]^

A prior systematic review reported that included 5 nonrandomized studies and only 1 randomized study for patients undergoing primary or emergent PCI, showed that delayed stent implantation is associated with better angiographic outcomes when compared to PCI with immediate stenting, but not with a reduction in major adverse cardiac events.^[8]^ Recent randomized trials have not consistently shown deferred stent implantation to be associated with improved clinical prognosis, angiographic outcomes, cardiovascular, or renal function.^[9–11]^ The value of delayed stenting in patients with STEMI and non-STEMI is largely unknown. Therefore, we aim to conduct this updated systematic review to assess whether delayed stenting is associated with improved angiographic and clinical cardiovascular outcomes.

## 2 Objectives

The objective of this study is to identify the effectiveness, safety, and long-term outcomes of different stenting strategy.

More specifically, the objectives are:

1. To assess the effectiveness of immediate versus deferred stenting in patients undergoing emergent or primary PCI (adverse angiographic outcomes, clinical outcomes, cardiovascular, and renal function);
2. To examine the effects of immediate versus deferred stenting on different subgroups (RCTs vs Non-RCTs, STEMI vs Non-STEMI, Older vs Non-Older, Diabetes vs Non-Diabetes).

## 3 Methods

### 3.1 Search strategy

Electronic databases search:

The following electronic databases will be searched systematically to identify relevant trials:

1. MEDLINE (Ovid);
2. Cochrane Central Register of Controlled Trials (Ovid);
3. EMBASE (Ovid).

Initial keywords to be used will be: Myocardial infarction; Percutaneous Coronary Intervention; Stent; Acute Coronary Syndromes; STEMI; Non-STEMI. We will apply the Cochrane sensitivity-maximizing RCT filter (Lefebvre 2011) to MEDLINE (Ovid). For EMBASE, we will use the multiterm EMBASE filter with the best balance of sensitivity and specificity (Wong 2006) translated from Ovid to embase.com syntax. We will search all databases from their inception to the present without any restriction on language of publication. If we detect additional relevant key words during any of the electronic or other searches, we will modify the electronic search strategies to incorporate these terms and document the changes.

Searching other resources:

In order to identify unpublished articles and those potentially missed through the electronic database searches, we will:

1. Hand search reference lists of all included studies and of relevant reviews retrieved by electronic searching to identify other potentially eligible trials or ancillary publications;
2. Hand search conference proceedings for the last 5 years from the following events: World Congress of Cardiology, European Society of Cardiology (ESC) Congress, ACC Annual Scientific Sessions, AHA Annual Scientific Sessions, and Transcatheter Cardiovascular Therapeutics Abstracts;
3. Attempt to contact the authors of trials when information in the study report is either lacking or unclear;
4. We will also search the following trial registers: ClinicalTrials.gov (www.clinicaltrials.gov).

### 3.2 Selection criteria for considering studies for this review

#### 3.2.1 Types of studies

We will include all randomized controlled trials (RCTs) and non-RCTS (registries, cohorts, etc.) comparing immediate versus deferred stenting strategy for myocardial infarction in people with STEMI and non-STEMI (with single or multi-vessel disease). This review will also consider experimental and epidemiological study designs, including non-RCTs, quasi-experimental, before and after studies, prospective and retrospective cohort studies, case–control studies, and cross-sectional studies as appropriate for inclusion.

#### 3.2.2 Types of participants

We will include adults aged 18 years and above, with myocardial infarction or ACS undergoing primary or emergent PCI, identified at the time of the index procedure.

#### 3.2.3 Types of intervention(s)

Patients presenting with STEMI or NSTEMI initially treated with primary PCI (in the case of STEMI) or emergent PCI (in the case of NSTEMI). The initial coronary angiography has to be performed before the treatment (immediate vs deferred stenting) was assigned.

*Types of outcomes*: This review will consider studies that include the following outcome measures:

##### 3.2.3.1 Primary outcomes

*Angiographic events*: Incidence of final post-PCI no-/slow-reflow, defined as absent flow (thrombolysis in myocardial infarction, TIMI grade 0), incomplete filling (TIMI flow grade 1), or slow-reflow but complete filling (TIMI flow grade 2) of the culprit coronary artery at the end of the PCI as revealed by the 1 or 2 coronary angiograms (in the case of deferred stenting strategy).

##### 3.2.3.2 Secondary outcomes

1. Long-term (≥6 months) composite of clinical outcome: All-cause mortality and major adverse cardiovascular events (MACE) including recurrent ACS (recurrent myocardial infarction, unplanned revascularization of the target vessel, etc.), hospital admission for heart failure or any other cardiovascular cause and measured with follow-up of more than 6 months postindex procedure.
2. Long-term all-cause mortality: defined as death from any cause with follow-up of more than 6 months postindex procedure.
3. In-hospital all-cause mortality.
4. Long-term recurrent ACS: nonfatal myocardial infarction, spontaneous myocardial infarction (will exclude periprocedural elevation of cardiac enzymes), unplanned repeat revascularization (either coronary artery bypass graft surgery or PCI) or any other recurrent ACS (unstable angina, etc.) and measured with follow-up of more than 6 months postindex procedure.
5. Long-term rehospital admission for heart failure or any other cardiovascular cause: defined as rehospital admission from heart failure or any other cardiovascular events with follow-up of more than 6 months postindex procedure.
6. Long-term stroke: defined as stroke from any cause with follow-up of more than 6 months postindex procedure.
7. Heart function improvement, myocardial salvage index as infarct size at 6 months indexed to the initial area at risk; incidence of reduced left ventricular ejection fraction; MVO, expressed as the relative percentage of total LV mass on the cardiac MRI done 3 to 8 days after the first procedure.
8. Contrast-induced nephropathy: defined as an increase (e.g., ≥25%, 50%, 0.3, 0.5) in plasma creatinine and measured more than 48 hours to 1 week postindex procedure.

*Comparator*: Immediate vs Delayed/Deferred.

### 3.3 Data collection and analysis

#### 3.3.1 Selection of studies

Two review authors (SMSI and YL) will independently screen titles and abstracts for inclusion of all the potential studies that we identify as a result of the search and code themas “retrieve” (eligible or potentially eligible/unclear) or “do not retrieve.” If there are any disagreements, we will ask a third review author to arbitrate (CKC or JC). We will retrieve the full-text study reports/publication and 2 review authors (SMSI and YL) will independently screen the full-text and identify studies for inclusion, and identify and record reasons for exclusion of the ineligible studies. We will resolve any disagreement through discussion or, if required, we will consult a third review author (CKC or JC). We will identify and exclude duplicates and collate multiple reports of the same study so that each study, rather than each report, is the unit of interest in the review. We will record the selection process in sufficient detail to complete a PRISMA flow diagram and “Characteristics of excluded studies” table.

#### 3.3.2 Data extraction and management

We will use a data collection form for study characteristics and outcome data that will be piloted on at least 1 study in the review. Two review authors (PC and KL) will extract study characteristics from included studies. We will extract the following study characteristics:

1. Methods: study design, total duration of study, details of any “run in” period, number of study centers and location, study setting, withdrawals and date of study.
2. Participants: number, mean age, age range, gender, comorbidities, severity of presenting STEMI OR Non-STEMI ACS, diagnostic criteria, inclusion criteria, and exclusion criteria.
3. Interventions: intervention, comparison, concomitant medications, and excluded medications.
4. Outcomes: primary and secondary outcomes specified and collected, and time points reported.
5. Notes: funding for trial and notable conflicts of interest of trial authors.

Two review authors (SMSI and YL) will independently extract outcome data from included studies using the standardized data extraction tool. We will resolve disagreements by consensus or by involving a third review author (CKC). One review author (CKC) will transfer data into the Review Manager 5 (RevMan 2014). We will double-check that data are entered correctly by comparing the data presented in the systematic review with the study reports. A second review author (JC) will spot-check study characteristics for accuracy against the trial report.

### 3.4 Quality assessment/assessment of risk of bias in included studies

Two review authors (SMSI and YL) will independently assess risk of bias for each study using the criteria outlined in the Cochrane Handbook for Systematic Reviews of Interventions (Higgins 2011). We will resolve any disagreements by discussion or by involving a third review author (CKC). We will assess the risk of bias according to the following domains (Wood 2008):

*RCTs using Cochrane methodologies*:

1. Random sequence generation.
2. Allocation concealment.
3. Blinding of participants and personnel.
4. Blinding of outcome assessment.
5. Incomplete outcome data.
6. Selective outcome reporting.
7. Other bias (e.g. industry funding).

*No-RCTs using Newcastle-Ottawa Scale*^[12]^:

Selection∗

1. Representativeness of the exposed cohort.
2. Selection of the nonexposed cohort.
3. Ascertainment of exposure.
4. Demonstration that outcome of interest was not present at start of study.
  Comparability
5. Comparability of cohorts on the basis of the design or analysis.
  Outcomes
6. Assessment of outcome.
7. Was follow-up long enough for outcomes to occur.
8. Adequacy of follow-up of cohorts.

We will grade each potential source of bias as high, low, or unclear and provide a quote from the study report together with a justification for our judgment in the “Risk of bias” table. We will summarize the risk of bias judgments across different studies for each of the domains listed. Where information on risk of bias relates to unpublished data or correspondence with a trialist, we will note this in the “Risk of bias” table. When considering treatment effects, we will take into account the risk of bias for the studies that contribute to that outcome.

#### 3.4.1 Assessment of bias in conducting the systematic review

We will conduct the review according to this published protocol and report any deviations from it in the “Differences between protocol and review” section of the systematic review. We will use the GRADE (Grades of Recommendation, Assessment, Development and Evaluation) approach to assess the quality of evidence related to each of the key outcomes listed in the Types of outcome measures (Chapter 12.2, Cochrane Handbook for Systematic Reviews of Interventions; Higgins 2011).

#### 3.4.2 Measures of treatment effect

We will analyze dichotomous data as odds ratios or risk ratios with 95% confidence intervals (CIs) and continuous data as mean difference or standardized mean difference with 95% CIs. We will enter data presented as a scale with a consistent direction of effect. We will describe skewed data reported as medians and interquartile ranges narratively.

#### 3.4.3 Unit of analysis issues

We will take into account the level at which randomization occurred, such as multiple observations for the same outcome. Considering the nature of the intervention, it is unlikely that we will find cross-over trials or cluster randomized trials. If these are found, we will take unit of analysis into account.

#### 3.4.4 Dealing with missing data

We will contact investigators or study sponsors to verify key study characteristics and obtain missing numerical outcome data where possible (e.g., when a study is identified as abstract only). Where this is not possible, and the missing data are thought to introduce serious bias, we will explore the impact of including such studies in the overall assessment of results using a sensitivity analysis.

#### 3.4.5 Assessment of heterogeneity

We will examine heterogeneity using the *I*^2^ statistic, which quantifies inconsistency across studies to assess the impact of heterogeneity on the meta-analysis, where an *I*^2^ statistic of 50% or more indicates a considerable level of inconsistency.

If we identify substantial heterogeneity, we will report it and explore possible causes by prespecified subgroup analysis and perform the analysis using a random-effects model. In the event of substantial clinical, methodological or statistical heterogeneity, we will not report study results as meta-analytically pooled effect estimates.

#### 3.4.6 Assessment of reporting biases

If we are able to pool more than 10 trials, we will create and examine a funnel plot to explore possible small study biases for the primary outcomes.

### 3.5 Data synthesis

Data analysis will be performed using RevMan software and STATA v13. Effect sizes expressed as odds ratio (for categorical data) and weighted mean differences (for continuous data) and their 95% CIs will be calculated. Heterogeneity will be assessed statistically using the standard Chi-square and also explored using subgroup analyses based on the different quantitative study designs included in this review. Where statistical pooling is not possible the findings will be presented in narrative form including tables and figures to aid in data presentation where appropriate. EndNote will be used for references.

If there is evidence for homogeneous effects across studies, we will analyze the data using risk ratios and summarize all data using a fixed-effect model (Riley 2011; Wood 2008). We will interpret fixed-effect meta-analyses with due consideration of the whole distribution of effects, ideally by presenting a prediction interval (Higgins 2011). In addition, we will perform statistical analyses according to the statistical guidelines contained in the Cochrane Handbook for Systematic Reviews of Interventions (Higgins 2011).

#### 3.5.1 Sensitivity analysis

We will perform sensitivity analyses in order to explore the influence of the following factors on effect sizes.

1. Restricting the analysis to published studies.
2. Restricting the analysis by taking into low versus high risk of bias, as specified in Assessment of risk of bias in included studies.
3. Restricting the analysis to studies using the following filters: language of publication, source of funding (industry vs other) and country.

## 4 Conclusions

This systematic review and meta-analysis will provide evidence and recommendations for clinical practice and management of patients with myocardial infarction. The implications of this research will suggest priorities for future research and outline what the remaining uncertainties are in the area.

### 4.1 Strengths and limitation of this study

- This meta-analysis will provide useful evidence-based guidance for interventional cardiologist to facilitate the clinical decisions when deciding on reperfusion strategies for patients undergoing primary or emergent percutaneous coronary intervention.
- The study will assess both the short-term angiographic outcomes (no or slow coronary flow after final PCI) and long-term clinical outcomes (e.g., mortality, recurrent ACS) improvement for delayed stenting (vs immediate stenting) among patients with STEMI or non-STEMI ACS.
- Several subgroup and sensitivity analyses will address relevant questions, such as patients with STEMI or non-STEMI ACS.
- Our results may be limited by the small size of the randomized trials and heterogeneity between trials, but will still address current uncertainties within the published literature.
